# Staphylococcus caprae: A Skin Commensal with Pathogenic Potential

**DOI:** 10.7759/cureus.3485

**Published:** 2018-10-23

**Authors:** Asha Gowda, Amanda L Pensiero, Clifford D Packer

**Affiliations:** 1 Internal Medicine, Case Western Reserve University School of Medicine, Cleveland, USA

**Keywords:** staphylococcus caprae, s. caprae, psoas abscess, bone infection, bone and joint infection, facet joint injection

## Abstract

Staphylococcus caprae (S. caprae) is a catalase-positive, coagulase-negative organism that was first isolated from goat milk, and was later found to colonize healthy human skin, nails, and nasal mucosa. Rarely, this commensal organism can become pathogenic in humans. S. caprae has been implicated in a variety of human infections, with the highest incidence being in bone and joint infections. We describe a man who, after receiving facet joint injections for back pain, developed native vertebral discitis, vertebral osteomyelitis with phlegmon, and bilateral psoas abscesses, from which S. caprae was isolated.

## Introduction

Coagulase-negative Staphylococcus (CoNS) species are recognized as part of healthy human skin flora. CoNS species are frequently encountered in clinical specimens as contaminants and, in general, are not considered to have the same pathogenic potential as the coagulase-positive Staphylococcus (S.) aureus [1]. The virulent properties of CoNS species are attributed to their ability to produce biofilm and colonize biomaterials [1]. Thus, when CoNS infections occur, they often can be resistant to many classes of antibiotics. We report the case of a man who, after receiving facet joint injections for back pain, developed native vertebral discitis, vertebral osteomyelitis with phlegmon, and bilateral psoas abscesses, from which S. caprae was isolated.

## Case presentation

A 74-year-old man with chronic back pain presented to the emergency department with a three-day history of worsening back pain that was unresponsive to multiple pain management regimens. Medical history was significant for end-stage renal disease (ESRD) requiring hemodialysis (HD) via his left internal jugular tunneled line, congenital alpha-2-antiplasmin deficiency, chronic pancytopenia, prostate cancer, hypertension, and hyperlipidemia. Two weeks prior to presentation, the patient was admitted for a one-month history of similar symptoms and was treated with fluoroscopy-guided corticosteroid injections of the L3, L4, and L5 vertebral facet joints bilaterally. Initially, he found relief; however, the pain returned after two weeks. On re-presentation, he had diarrhea and endorsed numbness of the right thigh but denied subjective fevers, chills, night sweats, or the recent use of antibiotics.

**Figure 1:**
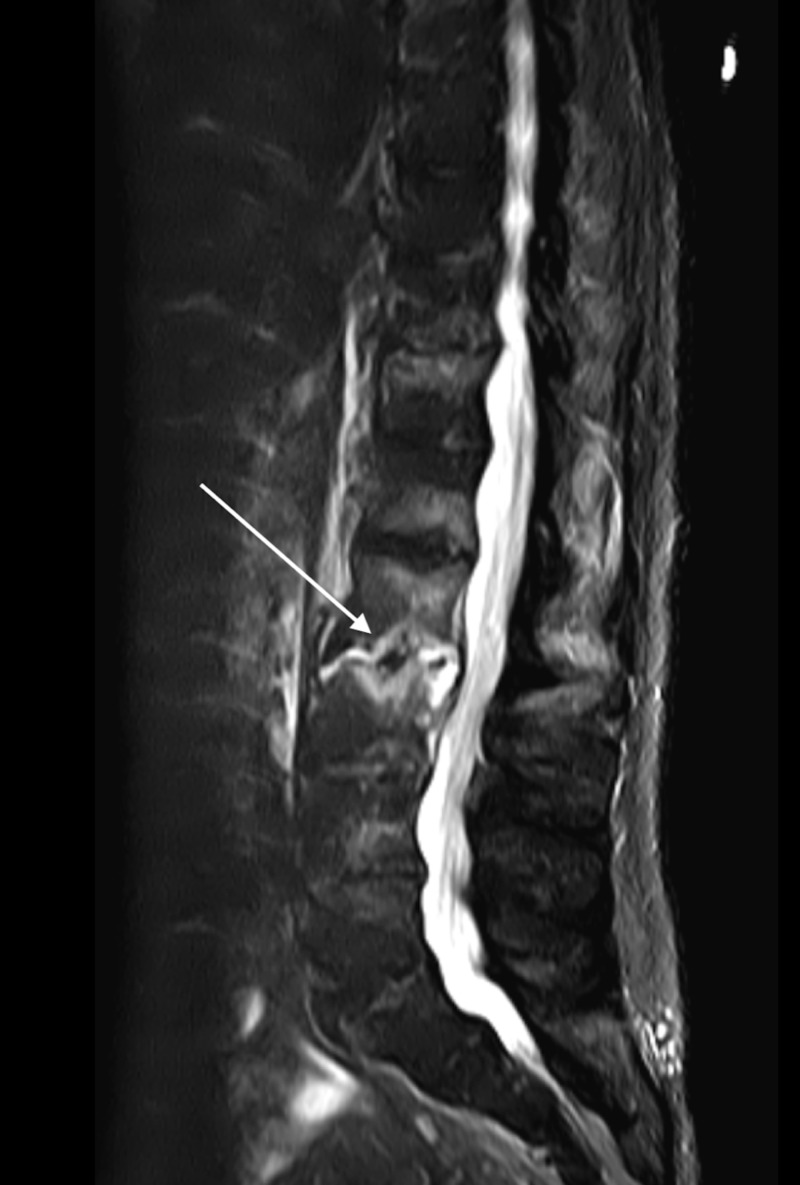
Magnetic Resonance Imaging with Contrast of the Lumbar Spine Sagittal image of the lumbar spine demonstrating acute discitis osteomyelitis with epidural phlegmon

**Figure 2:**
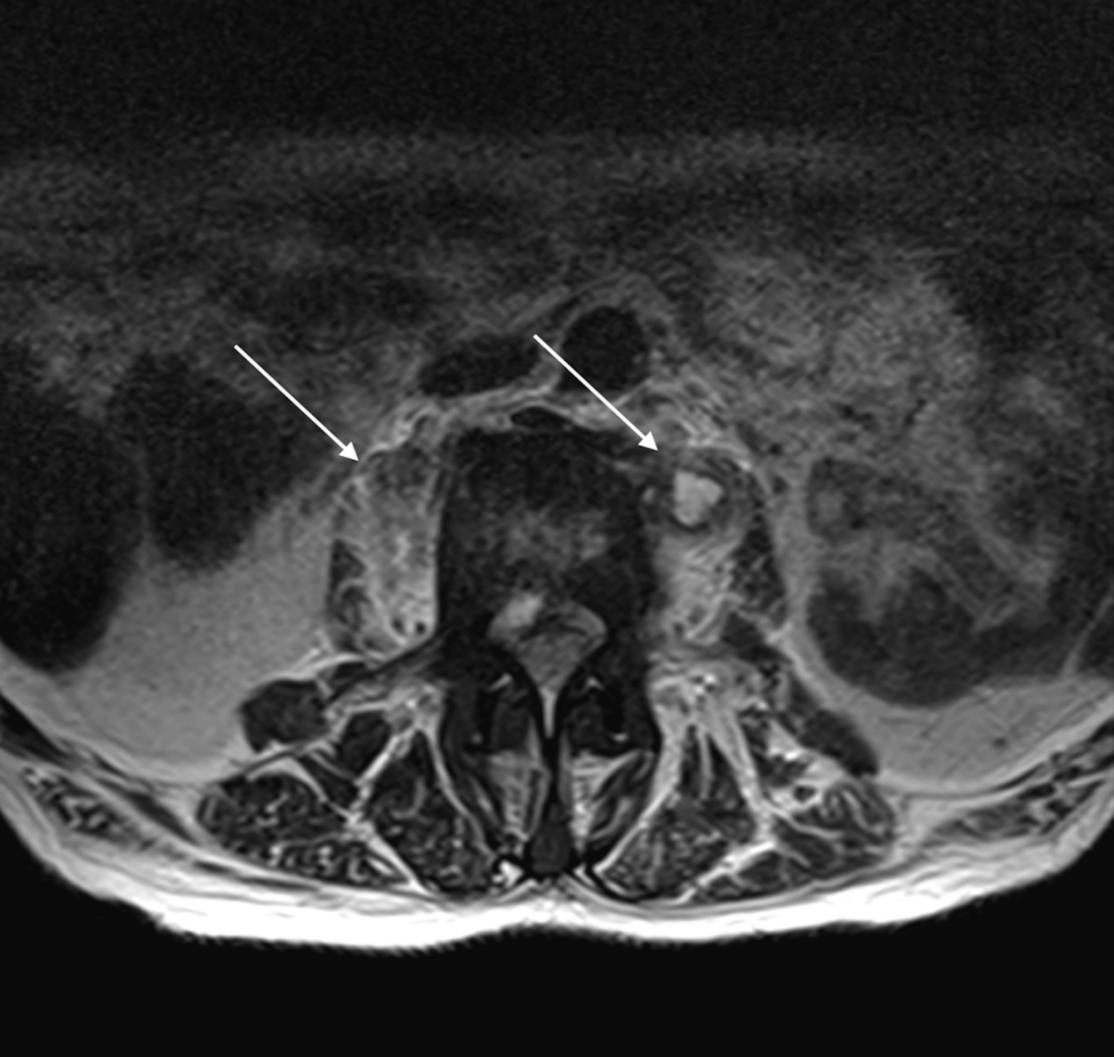
Magnetic Resonance Imaging with Contrast of the Lumbar Spine Axial image of the lumbar spine revealing multiple abscesses (see arrows) in the psoas muscles with prevertebral inflammation

The physical examination was significant for pain on deep palpation of the lower quadrants of the abdomen bilaterally and with light palpation over the lumbar spine and lumbar paraspinal muscles. Positive psoas signs were elicited bilaterally. The strength of the upper and lower extremities was preserved. Laboratory results were remarkable for pancytopenia (white blood cells 3.66 K/cmm, red blood cells 2.64 M/cmm, platelets 127 K/cmm) with neutropenia (1.38 K/cmm), elevated erythrocyte sedimentation rate (46 mm/hr), and C-reactive protein (116 mg/L). Magnetic resonance imaging (MRI) revealed the evidence of discitis at the L2-L3 vertebral joint, vertebral osteomyelitis with phlegmon, and multiple bilateral psoas abscesses (Figures 1-2).

**Figure 3:**
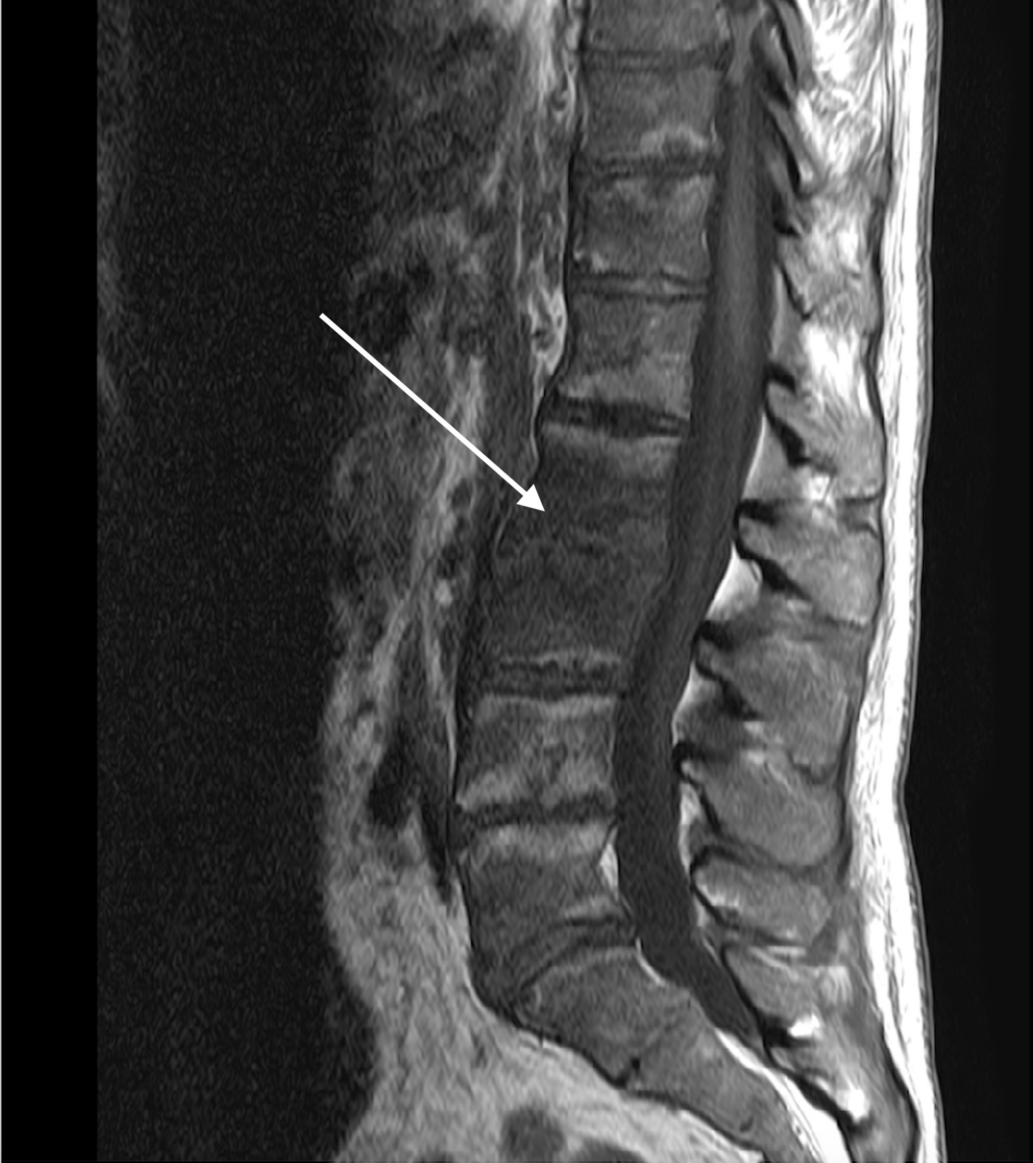
Magnetic Resonance Imaging Without Contrast of the Lumbar Spine Sagittal image revealing improvement of the acute discitis at the L2-L3 vertebral joint

**Figure 4:**
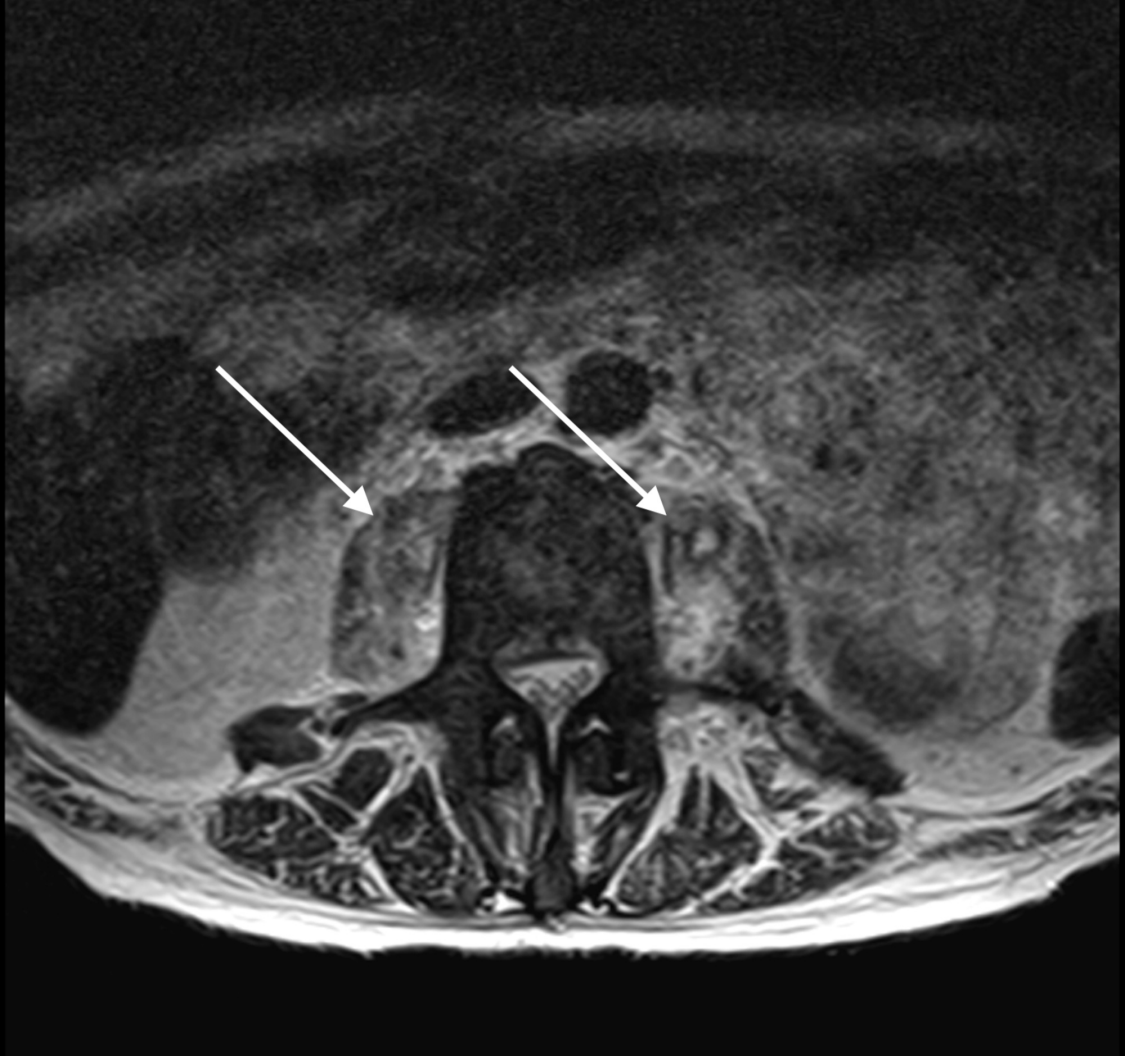
Magnetic Resonance Imaging Without Contrast of the Lumbar Spine Axial image of the lumbar spine revealing decreased size of the abscesses (see arrows) in the psoas muscles bilaterally

Cultures from the peripheral venous blood, hemodialysis (HD) line, and urine were obtained, and computed tomography (CT)-guided fine-needle aspiration of the abscesses yielded purulent fluid with gram-positive cocci on initial gram stain. The patient was empirically treated with renally dosed vancomycin and piperacillin-tazobactam after HD sessions. Cultures from the abscess fluid grew S. caprae and the treatment was narrowed to renally dosed cefazolin for six weeks. Several days later, the blood culture from the HD line also grew S. caprae, and the peripheral blood cultures yielded no growth. Therefore, his tunneled HD line was removed and after a 48-hour line holiday, a new tunneled dialysis catheter was placed. The urine culture yielded only normal urogenital flora at 48 hours. Transthoracic echocardiogram revealed no valvular vegetations and a normal systolic function. The patient’s back pain improved and he was discharged from the hospital. Follow-up magnetic resonance imaging (MRI) done 15 days later revealed small fluid collections in the psoas muscles and paraspinal soft tissue bilaterally that had regressed in size when compared to the prior imaging (Figures 3-4).

## Discussion

S. caprae is a catalase-positive CoNS species. This organism was initially isolated from goat milk in 1983 and is known to colonize the skin and mammary glands of goats [2-3]. Later, researchers isolated S. caprae in human specimens and considered it a colonizer of healthy human skin, nails, and nasal mucosa [2]. This organism is capable of infecting neonates, infants, and adults, however, infections in humans have been described infrequently [2]. The current literature describes the ability of S. caprae to behave as an opportunistic pathogen in immunocompromised hosts, causing both nosocomial and community-acquired infections. S. caprae has been isolated in cases of acute otitis media, acute otitis externa, mastoiditis, endocarditis, urinary tract infection, bacteremia, recurrent sepsis, meningitis, peritonitis, pneumonia, endophthalmitis, and, most frequently, bone and joint infections (BJI) [2-11]. The majority of reports describing S. caprae BJI implicates implanted orthopedic devices as the source of infection [2-4,9-11]. The frequent involvement of implanted materials is likely related to the ability of certain strains to form biofilm and adhere to surfaces through autolysin or fibronectin-binding proteins [10-14].

Although implanted biomaterials serve as a common route for nosocomial infection, native BJI with S. caprae occurs as well. S. caprae has been noted to produce slime, invade human epithelial cells, and exhibit cytotoxic effects, contributing to their virulence [10,12-14]. We reviewed 74 cases that described patients with S. caprae osteoarticular infections that have been published since 1997 [2,4,10-11,15-16]. Of these reports, 16 cases of S. caprae BJI occurred in the native bone and 54 occurred with devices. It was unclear whether an additional four were native infections. Of the 16 native BJI, only nine were definitely identified as monomicrobial infections. Aside from implanted devices, other risk factors for developing S. caprae BJI include recent antibiotic use, malignancies, diabetes mellitus, chronic renal failure, open fractures, immunodeficiency, and immunosuppression associated with systemic or local corticosteroids, chemotherapy, radiotherapy, and contact with sheep or goats [2,10,15]. Seng et al. also noted that 21 of their 25 patients with S. caprae infection were male, supporting a finding of a gender predilection that was previously made by Shuttleworth et al. [2,4]. An explanation for this observation remains unclear.

Our patient had comorbidities that increased his risk for an S. caprae infection, including ESRD and immunodeficiency, due to his chronic pancytopenia. Furthermore, any break in the skin, such as those created by injections, carries a small risk of infection. The corticosteroid injections given to our patient could have allowed for the direct inoculation of skin flora into deeper structures; in addition, the localized infusion of corticosteroid could have increased the risk for an opportunistic infection. Such an occurrence has been suggested by Seng et al., who described S. caprae BJI in two patients who received immunosuppressive and corticosteroid therapy for rheumatoid arthritis [2]. A nonsterile technique, skin contamination, or contamination of the injection needles also could have led to the iatrogenic infection. The growth of S. caprae from the preexisting HD line supports the likelihood of skin contamination, as seen commonly with CoNS species.

Although the normal skin flora of humans is made of multiple organisms, infections due to the inoculation of skin pathogens and hematogenous infections both tend to be monomicrobial in nature. Thus, it is not surprising that our patient had an infection with only this skin commensal. It is possible that the predominance of S. caprae in this case simply reflects the presence of this particular pathogen at the time of inoculation with the joint injections. Facet joint injections (FJI) with steroidal and/or anesthetic injections have been linked to several infectious complications. These include paraspinal muscle abscesses, cellulitis of the psoas muscle and soft tissues, epidural abscesses, meningitis, spondylodiscitis, subdural empyema, septic arthritis, endocarditis, and generalized infection leading to multiorgan failure and death [17-20]. The most impressive aspect of these reports is the microbial extension from the facet joint to neighboring areas. In one case, the tracking of the infection along the nerve root was clearly visualized on imaging [17]. S. aureus is the most frequently implicated organism in FJI-related infections in these cases, in addition to S. epidermidis and Pseudomonas aeruginosa [17-18,20]. It is reasonable to believe that like these organisms, infection with S. caprae after FJI also can extend to nearby structures, including the psoas muscle, vertebrae, and disc spaces. Other risk factors for post-FJI infections may include a history of diabetes mellitus, a concurrent local or systemic infection with subsequent seeding or extension, an immunocompromised state, or alcohol abuse [19]. An alternate theory of catheter-induced bacteremia was also considered in this case. This patient’s indwelling catheter used for HD, ESRD, and chronic pancytopenia with neutropenia may have increased his risk for S. caprae infection [2,5-6,15]. In addition to the catheter serving as a source of entry for the commensal S. caprae, the patient’s long-standing leukopenia increased his risk for bacteremia. There are reports of intravascular catheter-associated bacteremia with S. caprae, in one of which there was concurrent chemotherapy-induced neutropenia [5-6]. Given this organism's predilection for BJI, our patient’s multiple visits to the emergency department with complaints of back pain may have been due to a smoldering vertebral infection after pathogen entry. The joint injections with steroids likely created an environment that fostered the preexisting infection to grow and establish a niche in the psoas muscles, evidenced by positive cultures from the aspirated fluid. However, the growth of S. caprae from only the HD line and the negative cultures from the peripheral blood and catheter tip suggest that intravascular catheter-induced bacteremia and subsequent seeding to the vertebrae and psoas muscles were less likely.

## Conclusions

This report is the first to describe a patient who received facet joint injections and subsequently developed discitis, vertebral osteomyelitis with phlegmon, and psoas muscle abscesses with S. caprae as the implicated organism. The tendency of S. caprae to cause BJI is well-established, however, the isolation of S. caprae from muscle abscesses in humans has not been reported to date. Furthermore, we present this case to share the diagnostic challenges we encountered. We conclude that the direct inoculation of S. caprae due to skin contamination at the time of the corticosteroid injection was the likeliest mechanism of infection, although bacteremia cannot be completely ruled out.
